# A case of chorea-acanthocytosis with significant improvement of symptoms at one year with deep brain stimulation: case report and literature review

**DOI:** 10.3389/fneur.2024.1377377

**Published:** 2024-07-25

**Authors:** Yan Xu, Jiabin Yu, Yimeng Gao, Qiaozhen Su, Haitao Xie, Hongfeng Liang, Chunye Zheng

**Affiliations:** ^1^The Second Clinical College of Guangzhou University of Chinese Medicine, Guangzhou, China; ^2^Department of Neurology, Guangdong Provincial Hospital of Traditional Chinese Medicine, Guangzhou, China

**Keywords:** chorea-acanthocytosis, involuntary movement, abnormal vocalizations, globus pallidus internal, deep brain stimulation

## Abstract

Chorea-acanthocytosis (ChAc) is a rare, neurodegenerative disorder caused by mutations in the VPS13A gene. In this article, we report on a 32-year-old man diagnosed with ChAc, with involuntary movements of the mouth and trunk, drooling of the mouth, slurred speech, and abnormal vocalizations as the main clinical manifestations. Three weeks after implantation of globus pallidus internal (GPi)-deep brain stimulation (DBS), the patient’s symptoms improved significantly. For example, articulation is clear, involuntary trunk movements and salivation have largely disappeared, and abnormal vocalizations have been significantly reduced. After 1 year of follow-up, the improvement in involuntary movement symptoms is essentially the same as before. As far as we know, we are the first to report the relief of involuntary vocalizations in a patient with GPi-DBS treatment, and that salivation and involuntary trunk movements have almost disappeared, and all other symptoms are significantly relieved, which is rare in previous cases. All of the above proves that the treatment of our case with DBS was very successful and that longer term follow-up is critical. We also hope that our case will provide new references and therapeutic ideas for the future treatment of patients with ChAc.

## Introduction

Chorea-acanthocytosis (ChAc) is an extremely rare autosomal recessive neurodegenerative disorder caused by mutations in VPS13A, which is located on 9q21, and is a subtype of neuroacanthocytosis syndrome (1). Its main clinical features include progressive movement disorders, seizures, psychiatric symptoms, cognitive deficits, etc., and orofacial dyskinesia is one of its most prominent features, and there may be self-injurious behavior (2, 3). The prevalence of the disease is about 1: 1,000,000, and most patients with ChAc have elevated creatine phosphokinase (CK) levels, in addition to the fact that ChAc is mainly characterized by peripheral blood acanthocytosis (4). Magnetic Resonance Imaging (MRI) of the brain generally shows bilateral symmetrical atrophy of the caudate nucleus. Clinical treatment options for ChAc are currently greatly limited and only limited improvement from symptomatic and supportive therapy (5). Previous studies have found that GPi-DBS not only improves dystonia and choreiform symptoms in ChAc patients, but may also be the best option for ChAc patients with predominantly orofacial and submandibular dystonia (6–8). At the same time, we also reviewed the relevant literature to explore the characteristics and advantages of GPi-DBS treatment of ChAc, in hopes of creating a reference for future ChAc treatments.

## Case presentation

**Figure 1:**
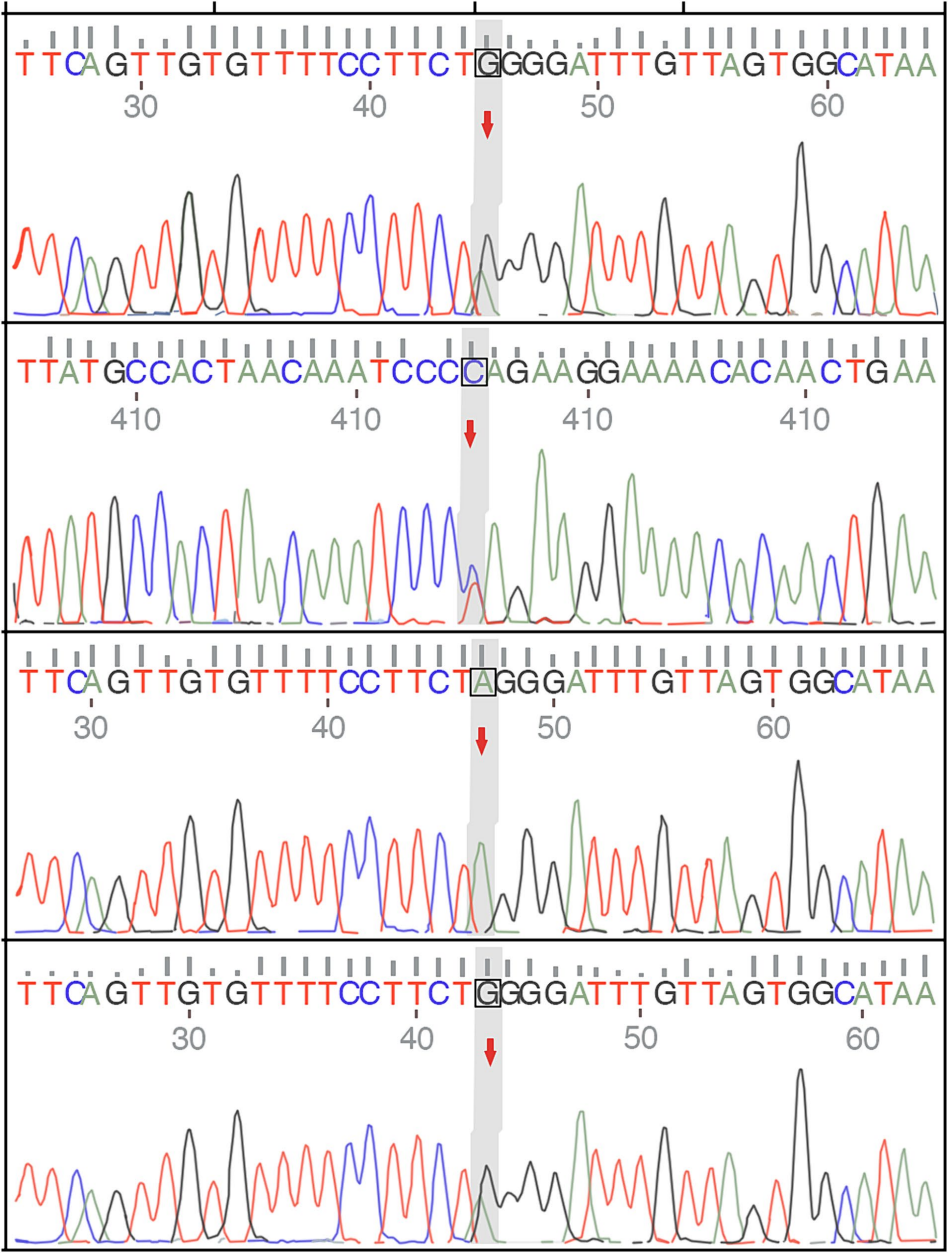
The genetic examination results of the patient and show a single nucleotide variant and a small fragment insertion–deletion variant.

A 32-year-old man presented in September 2022 with involuntary movements of the corners of the mouth, drooling of the mouth (noticeable during eating or drinking), frequent involuntary vocalizations (similar to grunting) slurred speech, occasional involuntary forward flexion, and an unsteady gait. The patient’s choreic symptoms had significantly worsened after 6 months, such as more frequent involuntary movements of the corners of the mouth and involuntary movements of the limbs, occasional involuntary forward flexion of the trunk with a slight tilt to the right when walking, and the number of abnormal vocalizations made increased. Moreover, he developed new symptoms, involuntary turning of the neck and shrugging of the shoulders, involuntary grimacing, and lacking fluency in swallowing. The patient had no other typical symptoms, such as cognitive, psychiatric, neuropathic, or myopathic symptoms or seizures. A physical examination of the patient revealed dysarthria, occasional choking on food or water, decreased sensation of the posterior pharyngeal wall bilaterally, reduced pharyngeal reflexes on both sides of the pharyngeal wall, slow elevation of the soft palate bilaterally in the region, a slight decrease in muscle tone in the extremities, and muscle strength of the limbs of Grade 5. Furthermore, his knee-jerk and achilles tendon reflexes were diminished. Laboratory tests indicated the presence of 9% acanthocytes in the blood smear, while serum ceruloplasmin levels were within the normal range at 284 mg/L (normal values range from 150–600 mg/L). The cranial MR suggests atrophy of the caudate nucleus on the left side of the cranium. According to the electromyogram results, abnormalities in the measured tibial nerve H-reflex suggest damage to the lumbosacral nerve root. Nerve conduction and electro-neurography of the remaining extremities showed no significant abnormalities. The genetic examination results (Figure 1) showed the patient had two heterozygous mutations in the VPS13A gene, mutation 1 (c.7253A > G, p.Asn2481Ser, source of variation: father) was located in the coding region: nucleotide 7,253 was mutated from adenine to guanine resulting in a mutation of amino acid 2,481 from asparagine to serine. Mutation 2 (c.8908-2A > G, source of variation: mother) was located at the classical splice site: the second nucleotide upstream of nucleotide 8,908 was mutated from adenine to guanine, and this mutation may result in aberrant splicing of the exon, which could lead to aberrant mRNA splicing affecting the normal structure and function of the translated protein product. And these mutations not were reported previously.

In the process of tracing the patient’s history, we found that there was no family history of neurological disease. In the past six months, the patient has tried a variety of drugs in other hospitals. In January 2023, the patient took tiapride hydrochloride (0.1 g/tid) and pramipexole (0.125 mg/tid). However, the symptoms did not improve. In one month, the patient accepted diazepam (5 mg/bid) and haloperidol (1 mg/bid), and the involuntary movements of the corners of the mouth began to improve, but as the patient continued to take the medication, the symptoms returned to the beginning. We tried treating the patient with levodopa and benserazide hydrochloride (0.125 mg/tid) after admission, but the symptoms did not improve. We also tried using Chinese medicine recipes for the patient. After taking Prof. Huang’s experienced Ge-Gen -Decoction, his drooling symptoms improved slightly, and other symptoms had not improved, but he wanted a greater improvement in the treatment effect. In the end, we recommend that patients try GPi-DBS therapy after careful consideration.

**Figure 2:**
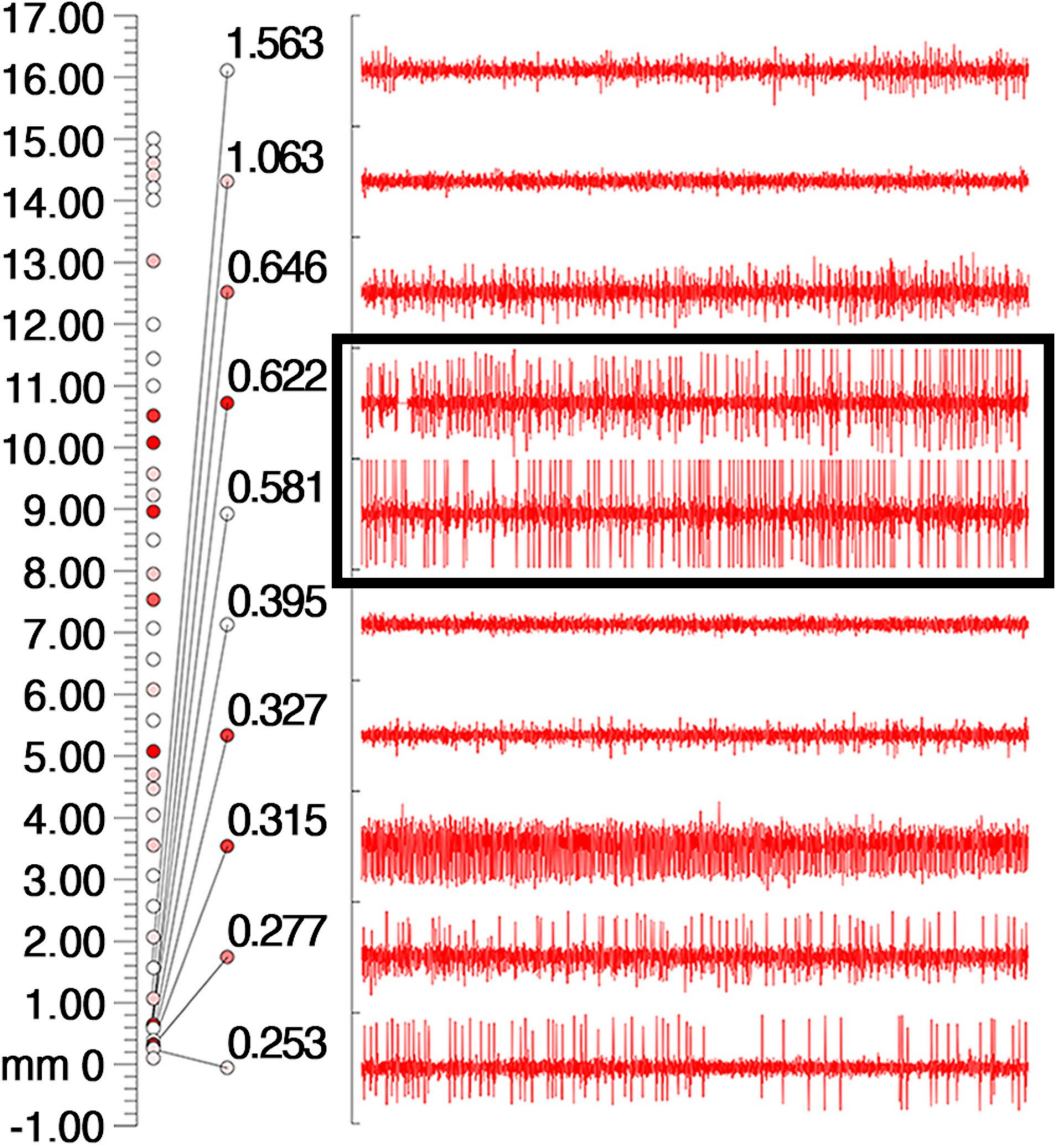
Intraoperative electrophysiologic monitoring unit observed cellular discharges typical of GPi. The coordinates on the leftmost side of the picture, the number 0 indicates the preset depth of the target point, the number 17 indicates 17 mm above the target point, and the cellular discharges are continuously recorded from the number 17 downwards, and the cellular discharges are the most typical and obvious in the black box, and then 0.622 mm and 0.531 mm above the target point. The different colored circles are related to the background sound (the sound during intraoperative testing of the nuclei), with the higher the background sound the redder the color.

**Figure 3:**
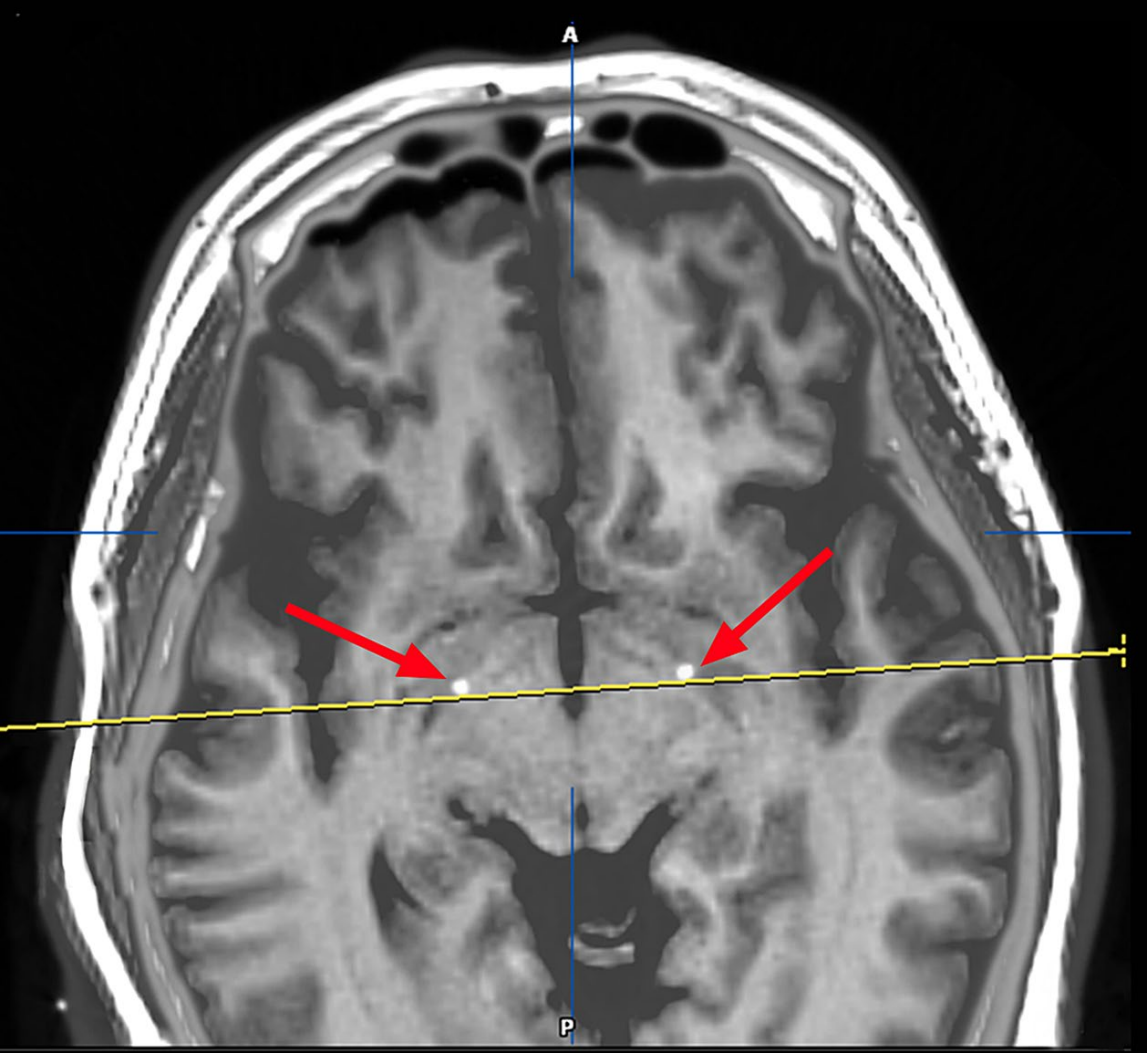
Image after fusion of preoperative MR plan and postoperative CT and it is a postoperative image fusion, suggesting that the stimulation electrode is precisely located in the GPI nucleus.

On March 23, 2023, we performed stereotactic deep brain electrode placement, as shown in Figure 2. In the first half of the procedure, deep brain electrodes are implanted while the patient is awake. Intraoperative electrophysiologic recordings of the GPi nuclei were satisfactory and in the correct position, and the location parameters are detailed in Table 1. After completing the first half of the procedure, we performed a Computed Tomography (CT) scan, fused the CT data with the preoperative implantation plan, and began implanting the peripheral pulse generator under general anesthesia after confirming the accuracy of the implantation position (Figure 3). We selected the corresponding parameters after a comprehensive evaluation based on the patient’s intraoperative symptom improvement and tolerance level (9). The frequency of the intraoperative test was 130 Hz, and the pulse width was 60 μs. In the third week after surgery (April 13, 2023), the patient returned to the hospital and DBS was turned on, and the stimulation parameters also started from low current and low frequency, and were slowly adjusted upward, while observing the patients’ symptom relief changes, and finally selecting the appropriate stimulation frequency and current (Table 1). Respectively, we assessed the patient’s pre-operative and post-operative Montreal Cognitive Assessment scores of 27 and 26(>26 normal cognitive functions). And we also assessed the patient’s pre-operative total Unified Huntington’s Disease Rating Scale (UHDRS) motor score of 53 and at post-operative week 3, a total UHDRS motor score of 21. After 1 year of follow-up, the improvement in involuntary movement symptoms is essentially the same as before. Ultimately, the patient’s choreic symptoms improved significantly, and a significant reduction in abnormal vocalizations.

**Table 1:** Target Coordinates and detailed stimulation parameters of the patient.

Location	X (mm)	Y (mm)	Z (mm)	Ring	Arc	Current	Pluse width (μs)	Frequency (Hz)
Left	122.8	99.5	101.0	54.7°	109.4°	2.0 V	120	135
Right	89.1	99.7	104.2	54.1°	75.4°	2.0 V	120	135

## Literature review

Through literature reviewed in the PubMed database from 1978 to 2023, a total of 35 patients treated with DBS were identified. In the end, 21 patients were included, as detailed in Table 2, after removing reports with grossly incomplete information or inaccessible literature. The first attempt to treat ChAc with DBS was made by Wihl et al. (21), however, the patients failed to benefit from DBS treatment. Since then, approximately 30 patients have been successfully treated with DBS for ChAc, with varying results and frequency of treatment. Significant remission of chorea and dystonia was achieved in all patients, with variable results for improvements in symptoms such as dysarthria and dysphagia. This could be related to different stimulus frequencies, pulse widths, or current intensities in individual patients.

**Table 2:** Summary of characteristics of ChAc patients with previously treated with DBS.

Time/Author	Age/Sex	VPS13A mutations	Main symptoms	Current	Pluse width (μs)	Fre (Hz)	UHDR (pre-op)	Follow-up (m)	UHDRS (post-op)	Outcome
2009/Ruiz et al. (10)	35/F	NA	Oromandibular dyskinesia, dysarthria, irregular choreic gait with truncal spasm	(L)4.4 V (R)4.2 V	180	130	24	NA	14	Rapid relief of tension disorder and chorea symptoms, dysarthria did not improved
2012/Shin et al. (11)	39/F	+	Whole body involuntary movement, orolingual dyskinesia with tongue protrusion and lip biting, involuntary head banging	(L)3.0 V (R)2.9 V	90	130	44	13	12	Dystonia, bradykinesia, choreic movements of the limbs and trunk had significantly improved, no improvement in chewing and biting of lips and tongue
2012/Li et al. (7)	39/M	NA	Orofacial and lingual dyskinesia, shoulder shrugging, neck stretching and tongue biting, chorea of the lower limbs and trunk	(L)3.5 V (R)3.5 V	60	40	36	9	13	Obvious improvement in chorea symptoms and dysphagia, mild improvements in dystonia with 40 Hz stimulation of the GPi, both chorea and dystonia were exacerbated by 130 Hz GPi stimulation.
	30/M	NA	Orofacial and lingual dyskinesia with tongue biting, generalized chorea and trunk spasms, difficulties in writing, speaking, swallowing, and walking	(L)3.5 V (R)3.5 V	60	40	53	5	27	
2013/Kefalopoulou et al. (12)	54/M	NA	Orofacial dyskinesias and feeding dystonia, violent trunk spasms	(L)2.5 V (R)2.5 V	60	130	40	NA	23	Upper limb dexterityand truncal dips improved markedly, no improvement in feeding dystonia
	43/M	NA	Oromandibular dyskinesias, dysarthria, violent choreic movements of upper and lower limbs, lurching gait and poor balance	(L)2.5 V (R)2.5 V	90	130	74	NA	50	Significant improvement in choreic movement, walking and truncal spasms
2015/Nakano et al. (13)	43/M	NA	Involuntary movements of the tongue and all extremities	(L)3.0–3.5 V (R)3.0–3.5 V	60–90	160	52	12	31	The dexterity of the upper extremities improved markedly
	40/M	NA	Dysarthria and oromandibular dyskinesia, progressive violent choreic movement of all extremities, trunk spasm and head-drop	(L)3.0–3.5 V (R)3.0–3.5 V	60–90	160	50	12	36	Decreased limb movement, dysarthria and slight head-drop did not improved
2015/Lee et al. (14)	36/M	+	Slurred speech, orofacial olingual dyskinesia, biting tongue and lip, and choreiform movements of the head and neck	(L)2.9 V (R)2.3 V	60	130	59	6	36–34	Marked improvement in choreic movements, biting tongue and lip
2016/Fernández-Pajarín et al. (15)	43/M	+	Abnormal movements of the orofacial region, upper limbs and trunk	(L)4 mA (R)4 mA	212	60	39	12	13	Marked improvement in orofacial dyskinesias and limb choreic movements
Author	Age/ Sex	VPS13A	Main Symptoms	Current	Pluse width (μs)	Fre (Hz)	UHDRS (pre-op)	Follow-up (m)	UHDRS (post-op)	Outcome
2018/Liu et al. (16)	35/M	+	Clenching and biting, dysphagia, slurred speech, involuntary head and limb	(L)2.6–3.5 mA (R)2.6–3.6 mA	70–100	150–165	62	12	18	Clinical improvement
	37/M	+	Biting, dysphagia, twitching limbs and trunk weakness and numbness of hands, myocardial infarction	(L)2.5–3.8 mA (R)2.5–3.3 mA	80–140	150–175	42	12	12	
	37/M	+	Biting, tongue involuntary movements, vocal tics, involuntary limb movements, generalized tonic–clonic seizure attack	(L)2.0–3.0 mA (R)2.0–3.0 mA	60–80	135–165	24	12	8	
	35/M	+	Biting, tongue involuntary movements involuntary limb movements, generalized tonic–clonic seizure attack	(L)2.5 mA (R)2.5 mA	90	160	22	12	8	
	36/F	+	licking lips, pouting, biting, dysphagia, chorea, gait instability	(L)2.0–2.7 mA (R)2.0–2.7 mA	90–100	150	48	12	14	Clinical improvement
	33/F	+	Biting, slurred speech, trembling legs and twitching arms, generalized tonic–clonic seizure attack	(L)0.0–1.6 mA (R)1.8–2.5 mA	60	130–145	16	12	6	
2018/Doshi et al. (17)	77/F	NA	Orofacial dyskinesias, neck dyskinesias	(L)3.0 V (R)3.0 V	90	130	36	NA	13	Dyskinesias were completely resolved
2019/Richard et al. (18)	31/M	+	Orolingual hyperkinesia, dysarthria, truncal and extremities chorea, gait instability, hyporeflexia, bradykinesia	(L)2.6 V (R)2.6 V	60	100	13	6	4	Speech, chorea and swallowing are ameliorated
2019/Wang et al. (19)	43/F	NA	Dysphagia, dysarthria, involuntary tongue protrusion, biting lip, and teeth grinding	(L)2.2 V (R)2.2 V	60	130	61	12	31	Significantly improvement in dysarthria, chorea and dyskinesia
2020/Wu et al. (20)	35/F	+	Involuntary movements of tongue, lower jaw, neck, trunk, and lower limbs, biting tongue and lip	(L)3.0 V (R)3.15 V	60	160	51	12	27	Remarkable remission in tongue biting, dysarthria, and gait abnormality
	35/M	+	Oromandibular dystonia, involuntary jaw movement, and upper limb right limb affecting handwriting.	(L)3.0 V (R)2.95 V	50	130	40	12	22	Remission of involuntary orofacial movements

NO., number; M, male; F, female; VPS13A, VPS13A mutations; Fre, frequency; Hz, hertz; NA, not available; UHDRS, unified Huntington’s disease rating scale score; pre-op, pre-operative; m, month; post-op, post-operative; +, yes.

## Discussion

Neuroacanthocytosis (NA) is a neurological syndrome associated with acanthocytosis, and its identification, diagnosis, and nomenclature have evolved. It was mainly used to characterize ChAc, McLeod syndrome (MLS), and Huntington’s disease-like 2 (HDL2) between 2001–2017, and it is understood that the current understanding of NA includes diseases caused by mutations in the VPS13A and XK genes (22, 23). However, there is a significant overlap in clinical symptoms, laboratory investigations, and imaging manifestations between the two subtypes. Genetic diagnosis is the primary method of differentiation. XK disease is a genetic disorder caused by mutations in the XK gene on the X chromosome that result in the loss or dysfunction of the encoded protein (24). The presentation of XK disease is characterized by muscular involvement, such as skeletal muscle myopathy, and peripheral neuropathy, and it can also affect other organ systems. Particularly, involvement of the heart may be a characteristic feature of the disease (25, 26).

ChAc is the most common type of neuroacanthocytosis. It may also be called VPS13A disease and is caused by pathogenic variants in the gene (27). The peak age of onset of the disease is 30–40 years of age, with progressive exacerbation of symptoms (28). Choreiform movements were the most prevalent clinical symptom in the disease (88%), followed by orofacial dystonia (80%), which includes increased facial expressions such as grimacing, smacking of the tongue, and biting of the tongue and lips (29, 30). Laboratory tests probably reveal an increase in the percentage of peripheral blood acanthocytes, which is a hint of the diagnosis of ChAc (31). Moreover, serum CK levels are significantly elevated. An Electromyogram is dominated by neuroaxonal damage and myopathic changes. Besides, cranial imaging may show symmetrical bilateral atrophy of the caudate. In this case, the patient was genetically tested and had classic choreic symptoms, so the ChAc diagnosis was clear.

At present, ChAc disease has no specific treatment, medications (such as dopamine blockers and VMAT2 inhibitors) can improve dyskinesia or rather chorea significantly, however not all of the symptoms of ChAc respond, and there may be side effects. Therefore, it is crucial to develop effective therapeutic approaches and evaluate treatment outcomes for ChAc patients. DBS is a widely used mainstream treatment for the treatment of movement disorders (9, 32). However, due to ChAc rarity, determining DBS therapeutic efficacy and safety remains challenging. A study was conducted to compare short- and long-term DBS treatment outcomes in fifteen ChAc patients, and the study found significant improvement in both chorea and dystonia, but results for dysarthria and swallowing were mixed. Additionally, akinesia did not improve among the patients (8). It is worth noting that we found most patients experience symptomatic relief with medium to high-frequency stimulation when summarizing the previous literature (Table 2), but Li et al. (7) reported two patients whose symptoms were relieved by low-frequency 40 Hz stimulation, on the contrary, both chorea and dystonia were exacerbated by 130 Hz GPi stimulation. We also found that most patients had pulse widths between 60 and 90 μs, with only two patients exceeding 150 μs and the stimulation current was between 2.5–3.0 volt (V) in most patients and 2.0–4.0 mA in some patients. The rational choice of stimulation frequency, pulse width, and current size for DBS treatment needs more research to verify, and we hope to make the treatment of the disease by DBS more standardized in the future.

In our case, the patient exhibited significant improvement in choreiform symptoms, It greatly improved the patient’s overall quality of life. To our knowledge, this is the first reported case of relief of involuntary vocalization symptoms through GPi-DBS treatment. Our patient had similar symptoms to a previously reported patient who experienced involuntary vocalizations (9). However, the patient in that report did not benefit from GPi-DBS stimulation. Fortunately, our patient responded well to the GPi-DBS treatment, with a reduction in involuntary vocalization, and movements of the trunk almost disappeared.

In conclusion, the patient’s symptoms have improved with DBS, particularly chorea, involuntary vocalization, and salivation at the corners of the mouth. It is reasonable to consider DBS as a safe treatment option for ChAc patients. However, the current reports on DBS for ChAc patients are mainly derived from a limited number of case reports and some small-sample retrospective studies from around the world. Therefore, further studies are still needed to confirm its efficacy and safety. Additionally, we believe that it is necessary to conduct long-term postoperative follow-ups of ChAc patients to continuously improve the treatment program and enhance their quality of life.

## Data availability statement

The datasets presented in this article are not readily available because of ethical and privacy restrictions. Requests to access the datasets should be directed to the corresponding authors.

## Ethics statement

Ethical review and approval was not required for the study on human participants in accordance with the local legislation and institutional requirements. Written informed consent from the patients/participants or patients/participants' legal guardian/next of kin was not required to participate in this study in accordance with the national legislation and the institutional requirements. Written informed consent was obtained from the individual(s) for the publication of any potentially identifiable images or data included in this article.

## Author contributions

YX: Investigation, Writing – original draft, Methodology. JY: Formal analysis, Methodology, Resources, Writing – original draft. YG: Data curation, Writing – original draft. QS: Conceptualization, Methodology, Resources, Writing – review & editing. HX: Methodology, Supervision, Validation, Writing – review & editing. HL: Conceptualization, Data curation, Validation, Writing – review & editing. CZ: Conceptualization, Resources, Writing – review & editing.
